# Shared psychosis at a distance: a case of telephone-induced Folie à Deux

**DOI:** 10.1192/j.eurpsy.2025.2208

**Published:** 2025-08-26

**Authors:** S. Castelao-Almodovar, A. Arce de la Riva, R. Albillos Perez, A. Pérez Balaguer, E. Gil Benito

**Affiliations:** 1 Centro de Salud Mental El Escorial, El Escorial; 2 H. U. Puerta de Hierro, Majadahonda, Spain

## Abstract

**Introduction:**

Shared psychotic disorder (*Folie à Deux*) is a phenomenon in which one person adopts the delusions of another with whom they have a close relationship. This case is particularly interesting because the delusions primarily developed through telephone conversations between two sisters and were notably exacerbated when they reunited in person. The telephone-based relationship between the sisters raises questions about the psychological influence from a distance in the development of shared psychosis.

**Objectives:**

To describe a clinical case of *Folie à Deux* in which delusion transmission occurred predominantly through telephone communication, highlighting the role of physical contact in the exacerbation of psychotic symptoms.

**Methods:**

We present the case of a 59-year-old woman hospitalized for shared psychosis. Her sister, with whom she maintained a close relationship through frequent phone calls, had previously developed persecutory delusions related to a complicated divorce. Over the course of five years, the patient began to share the same delusions of persecution and surveillance that her sister transmitted over the phone. However, following a visit from her sister to Madrid in July 2024, the patient’s psychotic symptoms intensified, leading to psychiatric hospitalization in the brief hospitalization unit (UHB), where antipsychotic and antidepressant treatment was initiated.

**Results:**

The patient was admitted with persecutory delusions centered on alleged surveillance related to her sister’s divorce, delusions that her sister initially developed and which they shared after years of phone conversations. During hospitalization, antipsychotic treatment was effective, leading to remission of the active psychotic symptoms. The patient demonstrated insight into her delusions, linking them to her sister’s influence. As contact with her sister decreased and treatment was introduced, the psychotic symptoms subsided. Since discharge, the patient has remained stable, though a medication adjustment was required due to reported side effects.

**Conclusions:**

This case of *Folie à Deux* highlights how a telephone relationship can be sufficient to transmit and maintain shared psychotic delusions. While physical contact exacerbated the symptoms, emotional exchange from a distance can also be a potent medium for perpetuating delusions. This case suggests that proximity, whether physical or emotional, directly influences the severity of shared psychosis.

**Disclosure of Interest:**

None Declared

